# Preparation and Self-Assembly of pH-Responsive Hyperbranched Polymer Peptide Hybrid Materials

**DOI:** 10.3390/nano13111725

**Published:** 2023-05-25

**Authors:** Yan Qin, Jianguo Yi, Yue Zhang

**Affiliations:** Hebei Key Laboratory of Functional Polymers, School of Chemical Engineering and Technology, Hebei University of Technology, Tianjin 300130, China; qy2137119427@163.com (Y.Q.); yjg892844210@163.com (J.Y.)

**Keywords:** hyperbranched polymer, polymer-peptide hybrid material, pH responsiveness, self-assembly, drug carrier

## Abstract

In recent years, the coupling of structurally and functionally controllable polymers with biologically active peptide materials to obtain polymer-peptide hybrids with excellent properties and biocompatibility has led to important research progress in the field of polymers. In this study, a pH-responsive hyperbranched polymer hPDPA was prepared by combining atom transfer radical polymerization (ATRP) with self-condensation vinyl polymerization (SCVP) using a three-component reaction of Passerini to obtain a monomeric initiator ABMA containing functional groups. The pH-responsive polymer peptide hybrids hPDPA/PArg/HA were obtained by using the molecular recognition of polyarginine (β-CD-PArg) peptide modified with β-cyclodextrin (β-CD) on the hyperbranched polymer, followed by the electrostatic adsorption of hyaluronic acid (HA). The two hybrid materials, h_1_PDPA/PArg_12_/HA and h_2_PDPA/PArg_8_/HA could self-assemble to form vesicles with narrow dispersion and nanoscale dimensions in phosphate-buffered (PB) at pH = 7.4. The assemblies exhibited low toxicity as drug carriers of β-lapachone (β-lapa), and the synergistic therapy based on ROS and NO generated by β-lapa had significant inhibitory effects on cancer cells.

## 1. Introduction

Compared with linear polymers, hyperbranched polymers with highly branched three-dimensional spherical structures have specific properties, such as better solubility, higher rheology, and abundant modifiable terminal groups [1,2,3,4]. Three methods are commonly used for the synthesis of hyperbranched polymers: (1) the ABx-type monomer condensation method; (2) the SCVP method; and (3) the ring-opening polymerization method. The SCVP method is the most commonly used method [5,6,7,8,9,10,11,12,13,14,15,16]. The method is based on the polymerization of a monomer initiator (inimer) containing both a double bond and an initiator group to prepare hyperbranched polymers [17,18,19,20,21,22]. It is widely used for its advantages, such as simple operation and mild reaction conditions, but the SCVP method also has some problems, such as a wide molecular weight distribution of the polymer and difficulty in controlling the molecular weight. The SCVP method is usually combined with reactive polymerization, such as reversible addition-fragmentation transfer polymerization (RAFT) or ATRP, to obtain structurally controlled hyperbranched polymers with a large number of RAFT chain transfer agents or ATRP initiators at their ends; for example, Patrickios and colleagues [23] prepared hydrophobic, degradable hyperbranched polymers PMMA by combining the SCVP method with ATRP, and obtained amphiphilic hyperbranched multi-arm polymers by using PMMA as a macromolecular initiator followed by polymerization of diaminoethyl methacrylate (DMAEMA). Subsequently, the ends can be modified to prepare functionalized polymers [24,25,26]. For example, Wais and colleagues [26] synthesized a series of star-shaped hyperbranched polymers by RAFT polymerization; these polymers were then functionalized by thiol-maleimide click reaction by changing the trithioester ends to sulfhydryl groups after an ammonolysis reaction. However, this process demands that a multi-step reaction be realized, so a monomer initiator with modifiable groups can be constructed by a multi-component reaction, and the functional hyperbranched polymers can be obtained by the SCVP-ATRP method using the monomer initiator. The peptides with biological functions are immobilized on polymers and can be applied in different industries such as medicine, the food industry, and functional carriers [27,28,29,30].

Hyaluronic acid (HA) is a negatively charged polysaccharide formed by (1-β-4)-glucuronic acid and N-acetylglucosaminoglycan disaccharide as repeating units, with an average molecular weight between 10^3^ and 10^7^ Da [31]. In 1934, HA was first purified from the vitreous humor of bovine eyes by Meyer, an American ophthalmology professor [32]. Due to its negative electrical charge, HA can form conjugates with positively charged compounds by electrostatic interaction. For example, Lenormand and colleagues [33] formed conjugates of HA and bovine serum protein (BSA) by electrostatic interaction at pH = 4 and found that different molecular weights of HA had a significant effect on the structure of the conjugates. Natural macromolecules are renewable resources and have good biocompatibility, and they can also shield positive charges by electrostatic adsorption, thus reducing cytotoxicity. Consequently, the use of natural macromolecules to form polymer conjugates has potential applications in drug carriers, biomedicine, cosmetics, and food products [34,35,36].

**Scheme 1:**
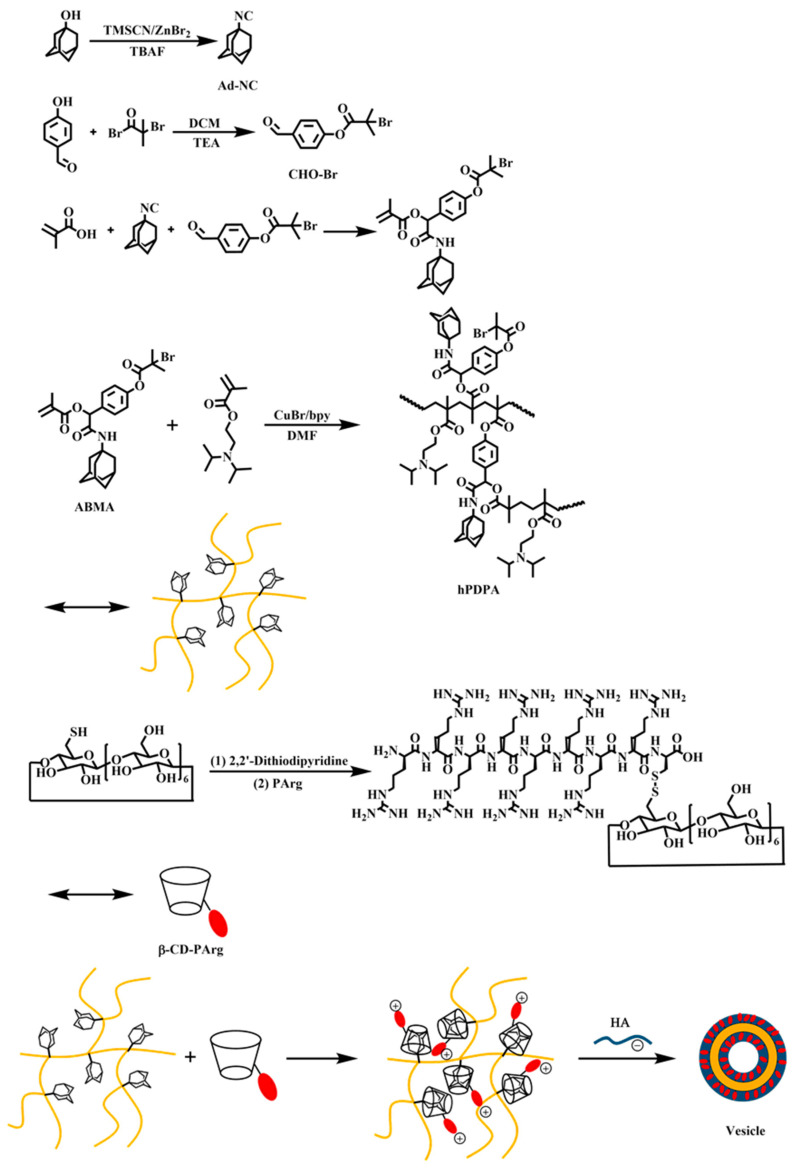
Synthetic route of hyperbranched polymer peptide conjugates.

In this paper, a pH-responsive hyperbranched polymer hPDPA was synthesized by combining SCVP with ATRP, and a pH-responsive hyperbranched polymer peptide conjugates hPDPA/PArg was obtained using the recognition of adamantane groups on the hyperbranched polymer with β-CD-PArg by host-guest interaction. Then, the hyperbranched polymer peptide hybrid material hPDPA/PArg/HA was obtained by adsorption of HA through electrostatic interaction. The self-assembly behavior of hPDPA/PArg/HA with different branching degrees and the application of the assemblies as drug carriers for β-lapachone (β-lapa) were investigated. Both polymer peptide hybrid materials, h_1_PDPA/PArg_12_/HA and h_2_PDPA/PArg_8_/HA were found to self-assemble into vesicles in PB at pH = 7.4, and β-lapa can be effectively loaded by vesicles and released under acidic conditions (pH = 5.0) (Scheme 1). The carriers h_2_PDPA/PArg_8_/HA showed low cytotoxicity towards Hela cells. The release of β-lapa leads to an increase in reactive oxygen species, which can react with PArg to generate NO. The reaction between reactive oxygen species and NO leads to the production of strongly cytotoxic peroxynitrite (ONOO^−^), and this synergistic therapy based on ROS and NO has a significant inhibitory effect on cancer cells.

## 2. Materials and Methods

Amantadine alcohol (Heowns, Tianjin, China, 97%). ZnBr_2_ (Aladdin, Tianjin, China, 98%). Trimethylsilyl cyanide (TMSCN, Heowns, Tianjin, China, 97%). Tetrabutylammonium fluoride solution (TBAF, Aladdin, 1.0 M in THF, Tianjin, China). p-hydroxybenzaldehyde (Heowns, Tianjin, China, 99%). 2-Bromoisobutyryl bromide (Heowns, Tianjin, China, 95%). Methacrylic acid (Sigma Aldrich, Tianjin, China, 99%). Diisopropylaminoethyl methacrylate (DPA, Aladdin, Tianjin, China, 98%). 2,2′-Dithiodipyridyl mono(6-mercapto-6-desoxy)-β-cyclodextrin (β-CD-SH, Zhiyuan Biotechnology Co. Ltd., Shandong, China, 98%). Polyarginine (PArg, Gill Biochemical Co. Ltd., Tianjin, China). Hyaluronic acid (HA, Melun Biological, Tianjin, China, 99%). β-Lapachone (β-lapa, Aladdin, Tianjin, China, 99%). All solvents were distilled after desiccant drying prior to use.

### 2.1. Synthesis of 1-Isocyanadamantane (Ad-NC) [37]

Amantadine alcohol (658 mg, 4.33 mmol), ZnBr_2_ (2.95 g, 13.1 mmol), and 25 mL CH_2_Cl_2_ were added to a 100 mL round-bottom flask, the oxygen was removed by bubbling with argon gas, and TMSCN (1.60 mL, 13.2 mmol) was added rapidly under the atmosphere of argon gas. The reaction was confined at room temperature for 18 h. Then, 13 mL TBAF solution was added and stirred for 20 min. The reaction solution was washed three times with a saturated NaHCO_3_ solution; then, the aqueous phase was extracted with ether. The combined organic phase was washed three times with a saturated NaCl solution and ultrapure water and finally dried with MgSO_4_; then it was separated by column chromatography with petroleum ether: ethyl acetate = 16:1. The separation of Ad-NC was obtained by column chromatography (yield: 60%).

### 2.2. Synthesis of 4-Formylphenyl-2-bromo-2-methylpropionate (CHO-Br) [38]

P-hydroxybenzaldehyde (972 mg, 7.97 mmol) was dissolved in 15 mL CH_2_Cl_2_, triethylamine (TEA, 1.40 mL, 10.1 mmol) was added, and the reaction was placed in an ice-water bath. Dibromoisobutyryl bromide (1.20 mL, 9.71 mmol) was dissolved in 5 mL CH_2_Cl_2_ and added dropwise to the above solution. The reaction was stirred for 12 h at room temperature. The reaction solution was washed three times with saturated NaHCO_3_ solution and ultrapure water and dried with MgSO_4_; then it was separated by column chromatography with petroleum ether: ethyl acetate = 6:1. The separation of CHO-Br was obtained by column chromatography (yield: 70%).

### 2.3. Synthesis of 2-((Adamantan-1-yl)amino)-1-(4-((2-bromo-2-methylpropanoyl)oxy)phenyl)-2-oxoethyl Methacrylate (ABMA)

Ad-NC (127 mg, 0.789 mmol), CHO-Br (107 mg, 0.399 mmol), and methacrylic acid (39.0 mg, 0.542 mmol) were dissolved in 1 mL CH_2_Cl_2_, and the reaction was stirred at room temperature for 48 h. The crude product was purified by silica gel column chromatography, petroleum ether: ethyl acetate = 12:1. (Yield: 70%)

### 2.4. Synthesis of Hyperbranched Poly(diisopropylaminoethyl Methacrylate) (hPDPA)

2,2′-bipyridine (bpy, 67.1 mg, 0.429 mmol) was dissolved in 0.6 mL DMF in a 5 mL Schlenk flask and degassed by three cycles of freeze-pump-thaw. CuBr (20.5 mg, 0.143 mmol) was added rapidly under argon atmosphere, the flask was degassed by three cycles of freeze-pump-thaw and was stirred under argon atmosphere at room temperature for 0.5 h. The monomer initiator ABMA (123 mg, 0.239 mmol) and DPA (300 mg, 1.41 mmol) were dissolved in 0.5 mL DMF in another 5 mL Schlenk flask and degassed by three cycles of freeze-pump-thaw. 0.5 mL CuBr solution was transferred into the polymer solution under argon protection, and the mixture was degassed by three cycles of freeze-pump-thaw. The polymerization was stirred at 60 °C for 5 h. After the polymerization reaction, DMF was removed from the reaction system, and the copper salts were removed by a neutral alumina column using CH_2_Cl_2_ as a drenching agent; the polymer was dissolved in a small amount of DMF and dialyzed to obtain h_1_PDPA. The details of the hyperbranched polymers are shown in Table 1.

### 2.5. Synthesis of β-Cyclodextrin-Polyarginine (β-CD-PArg)

To synthesize β-CD-PArg, β-CD-dithiodipyridine (β-CD-s-s-py) was first synthesized by a reaction between β-CD-SH and 2,2′-dithiodipyridine (py-s-s-py). β-CD-SH (400 mg, 0.348 mmol) and py-s-s-py (230 mg, 1.05 mmol) were dissolved in 8 mL DMF, and the reaction was conducted at room temperature for 24 h. The product was purified by precipitation in acetone and centrifugation. The upper layer of yellow liquid was washed with acetone until the upper layer of liquid was colorless, and the precipitate was the target product.

β-CD-s-s-py (80.0 mg, 0.0651 mmol) and PArg (37.0 mg, 0.0270 mmol) were dissolved in 2.2 mL of DMF (containing 0.2 mL ultrapure water), and the reaction was conducted at room temperature for 24 h. The crude product was purified by precipitation in acetone, centrifuged, and the supernatant was taken to obtain β-CD-PArg.

### 2.6. Synthesis of Polymer Peptide Conjugates hPDPA/PArg/HA

The hyperbranched polymer hPDPA was dissolved in DMF, β-CD-PArg was dissolved in DMSO and mixed in a certain ratio, and HA was dissolved in 1 mL of pH = 7.4 phosphate buffer solution (PB, 10 mM), and 0.2 mL of the mixed solution was slowly added dropwise to the HA solution. The assemblies were obtained by dialysis after rapid stirring for 2 h. The details of the assembly of the two polymer peptide hybrids are shown in Table 2.

### 2.7. Synthesis of hPDPA/PArg/HA-β-lapa

The polymer peptide conjugates hPDPA/PArg/HA was first synthesized. A total of 2.0 mg β-lapa was dissolved in 0.5 mL DMSO, and 0.062 mL β-lapa solution was added dropwise to the above solution; stirring continued for 12 h. After the encapsulation process was completed, the unencapsulated β-lapa was removed by dialysis and centrifugation. The nanoparticle powder encapsulated with β-lapa was dissolved in DMSO and detected by UV-Vis spectrophotometer, and the encapsulation rate (EE) and drug loading rate (LC) of β-lapa were calculated according to the formula:

$$EE\left(\%\right)=\frac{m_{b}}{m_{o}}\times 100\%$$

$$LC\left(\%\right)=\frac{m_{b}}{m_{b}+m_{p}}\times 100\%$$

where the *m_b_* is the mass of the encapsulated β-lapa, *m_o_* is the original β-lapa mass, and *m_p_* is the mass of the polymer hybrid material.

### 2.8. In Vitro Release of β-lapa from h_2_PDPA/PArg_8_/HA-β-lapa

The assembled solution of h_2_PDPA/PArg_8_/HA-β-lapa was added to PB at different pH values and placed at 37 °C with constant stirring. 1 mL solution was lyophilized and dissolved in 1 mL DMSO every two hours, and the absorption intensity was measured by UV-Vis spectrophotometer to calculate the amount of released β-lapa according to the equation, and then supplemented with 1 mL PB to continue stirring.

### 2.9. In Vitro Cytotoxicity Assays

h_2_PDPA/PArg_8_/HA and h_2_PDPA/PArg_8_/HA-β-lapa were cultured with Hela cells, respectively, and the cytotoxicity of hyperbranched polymer peptide hybrid materials was tested using the CCK-8 method. Hela cells were inoculated into 96-well plates with 5 × 10^3^ cells per well. The cells were incubated in the medium of DMEM with 10% FBS at 37 °C and 5% CO_2_ for 12 h until the cells were stable. h_2_PDPA/PArg_8_/HA and h_2_PDPA/PArg_8_/HA-β-lapa at different dilution concentrations were added to the medium and incubated for 24 h, respectively, and then the medium was carefully removed, and 100 μL of the medium was added to each well again (containing 10 μL CCK-8 reagent) and continued to incubate in a constant temperature incubator at 37 °C for 1 h. The absorbance at 450 nm was measured using an enzyme marker. Cell viability was calculated by using the following formula:

$$Cell\;viability\left(\%\right)=\frac{As-Ab}{Ac-Ab}\times 100\%$$

where *As* and *Ac* represent the absorbance of the cells treated by h_2_PDPA/PArg_8_/HA-β-lapa and the control cells (untreated), respectively. *Ab* is the absorbance of CCK-8 regents without cells.

### 2.10. Intracellular NO, ROS, and ONOO^−^ Release Assay

Hela cells were inoculated onto 35 mm^2^ confocal-specific dishes with 1 × 10^5^ cells per well. The cells were incubated at 37 °C with 5% CO_2_ for 12 h. Diluted h_2_PDPA/PArg_8_/HA and h_2_PDPA/PArg_8_/HA-β-lapa were added, respectively, and the culture medium was removed, washed with PBS, and fixed with 4% cell tissue fixative for 10 min, then washed again with PBS, and the dilutions were provided according to the kit. The NO probe (DAF-FM DA probe) was diluted, 1 mL of diluted DAF-FM DA was added to each well, incubated in a 37 °C cell incubator for 20 min, then stained with DAPI for 5 min, observed by laser confocal microscopy (CLSM) and photographed. ROS were detected using the DCFH-DA probe and ONOO^−^ using the O72 probe.

## 3. Results and Discussion

In this study, the monomer ABMA is a key compound for the synthesis of hyperbranched polymer hPDPA. It was synthesized by the Passerini three-component reaction of Ad-NC, CHO-Br, and methacrylic acid, providing the polymer branched chains with bromo groups to initiate the polymerization and adamantyl groups for molecular recognition. According to a previous report, Ad-NC was synthesized directly from adamantanol in one step. The synthesis of Ad-NC was confirmed by ^1^H NMR and ^13^C NMR spectra (Figure S1, Supporting Information). The ^13^C NMR spectrum of Ad-NC exhibits a peak at 150.66 ppm, belonging to the isocyanine group. CHO-Br was obtained by the esterification reaction of p-hydroxybenzaldehyde and 2-bromoisobutyryl bromide. The ^1^H NMR spectrum of CHO-Br is shown in Figure S2. The synthesized ABMA was obtained in high yield (70.1%), and due to the high reactivity of the isocyanate, the reaction conditions were mild. As shown in the ^1^H NMR spectrum of ABMA (Figure S3, Supporting Information), the characteristic peaks of products of the Passerini reaction appear at 5.74 and 5.95 ppm, corresponding to the protons of the newly formed tertiary carbon atom and the amide group, respectively. In the ^13^C NMR spectrum (Figure S3), the peak at 74.03 ppm can be observed for the newly formed tertiary carbon atom. The NMR spectrum results can prove the successful preparation of monomer ABMA.

Two hyperbranched polymers, hPDPA with different branching degrees but similar molecular weights, were obtained by adjusting the feeding ratios of monomer initiator ABMA and copolymer monomer DPA. h_1_PDPA and h_2_PDPA were obtained with feeding ratios of 6 and 10 for DPA and ABMA, respectively. h_1_PDPA and h_2_PDPA were characterized by ^1^H NMR and GPC.

**Figure 1:**
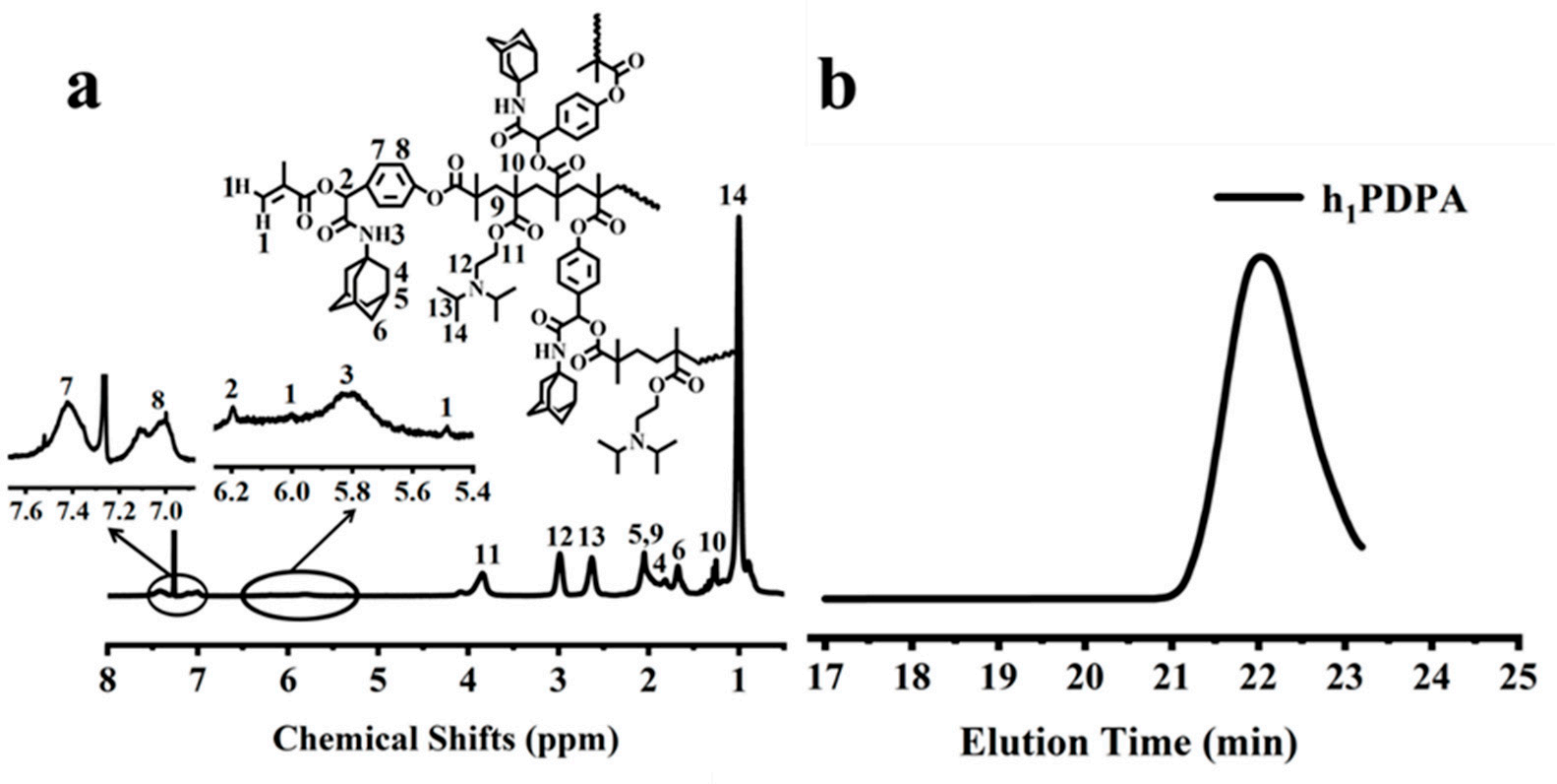
(**a**) ^1^H NMR spectrum in CDCl_3_; (**b**) GPC curves of h_1_PDPA.

The ^1^H NMR spectrum of the hyperbranched polymer h_1_PDPA is shown in Figure 1a. As can be clearly seen, the peaks at 7.47 ppm and 7.20–6.91 ppm correspond to protons on the phenyl group of the monomer initiator ABMA, and the peaks at 6.20 ppm and 5.81 ppm correspond to protons on the tertiary carbon atom and on the amide, respectively, which are characteristic peaks formed by the Passerini reaction. The peaks at 3.85 ppm and 3.11 ppm correspond to methylene protons on the copolymer monomer DPA, and the peaks at 2.77 ppm and 1.20 ppm correspond to protons on the tertiary carbon atom and on the terminal methyl group of the copolymer monomer DPA, respectively. The peak intensity of the proton on adamantine at 2.10 ppm is higher due to the inclusion of protons on the main chain methylene group. The ^1^H NMR spectra of the hyperbranched polymer h_2_PDPA are shown in Figure S4. Based on the ^1^H NMR results, the molecular weight of the hyperbranched polymer, the degree of polymerization (DP_n_) of the two monomers, and the ratio can be obtained by integrating the characteristic peaks. The DP_n_ ratio of DPA to ABMA for h_1_PDPA and h_2_PDPA was calculated to be 5.8 and 11.2, and the obtained polymerization ratio was close to the feeding ratio. As the feeding ratio of DPA to ABMA increased, the percentage of monomer initiator ABMA decreased, and the branching degree gradually decreased. The GPC curve of h_1_PDPA is shown in Figure 1b. It can be seen that the GPC curve shows a single-peak distribution, as the hyperbranched polymers are all nitrogenous compounds, which will adsorb to the column, making the peak emergence time late and incomplete. The same phenomenon can be observed in the GPC curve of h_2_PDPA (Figure S5). The structural data of the hyperbranched polymers based on ^1^H NMR and GPC results are summarized in Table 3.

**Figure 2:**
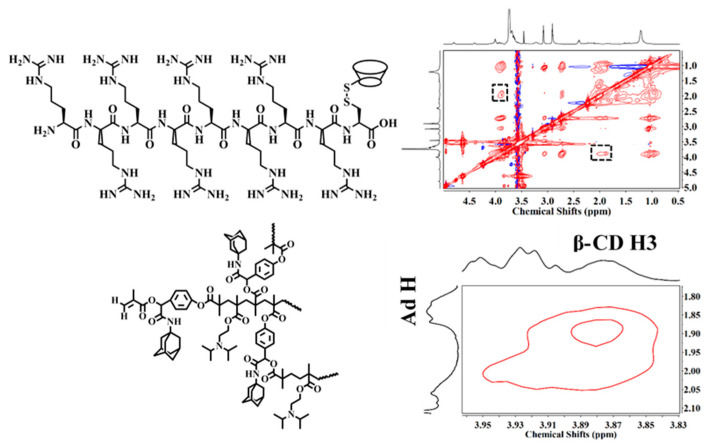
2D ^1^H NOESY NMR spectrum of the inclusion complex h_1_PDPA/β-CD-PArg.

According to Table 3, the number of adamantane groups of h_1_PDPA and h_2_PDPA can be obtained, which are 12 and 8, respectively. Therefore, it can be functionalized by recognition with molecules containing β-CD. In this study, β-CD-PArg was used as a model peptide for interaction with hyperbranched polymers. β-CD-s-s-py was prepared according to a previous report [39], and its ^1^H NMR is shown in Figure S6. The β-CD-PArg was obtained by a disulfide bond exchange reaction between the sulfhydryl group and β-CD-s-s-Py, and the ^1^H NMR of PArg before and after modification is shown in Figure S7. 2D NMR spectroscopy is a common method for analyzing the host-guest interaction because the H3 of cyclodextrin is located in the hydrophobic inner cavity, and when the adamantane group enters the inner cavity, the interaction with H3 is enhanced so that cross peaks appear on the 2D NMR spectra [40]. In this study, Host-guest recognition of β-CD-PArg and hyperbranched polymer hPDPA (in molar amounts of adamantane groups) in a mixture of DMF and DMSO. The 2D NOESY NMR spectra of the hyperbranched polymer h_1_PDPA and β-CD-PArg are shown in Figure 2, with chemical shifts showing cross peaks at 1.80–2.05 ppm and 3.84–3.95 ppm, indicating that the adamantane group is incorporated into the hydrophobic cavity of β-CD.

**Figure 3:**
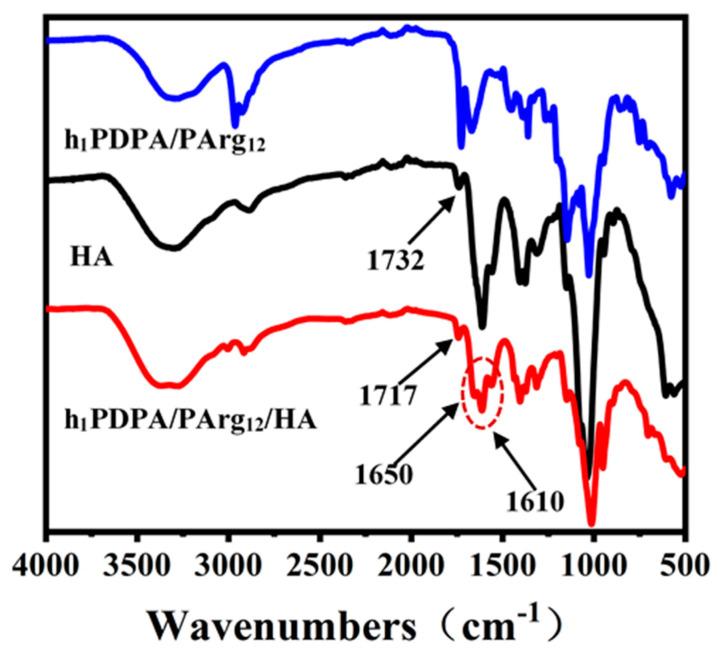
FTIR spectra of HA (black line), h_1_PDPA/PArg_12_ (blue line), and h_1_PDPA/PArg_12_/HA (red line).

Due to the positively charged nature of peptides, they can adsorb HA by electrostatic interaction, and infrared spectroscopy is a common characterization method to study the formation of complexes between two substances by electrostatic interaction [41,42]. The infrared spectra of HA, h_1_PDPA/PArg_12_, and h_1_PDPA/PArg_12_/HA are shown in Figure 3, in the spectrum of HA, the absorption peak at 1732 cm^−1^ belongs to the stretching vibration absorption peak of C=O (COOH), while the spectrum of h_1_PDPA/PArg_12_/HA shows this absorption peak shifted right to 1717 cm^−1^ and weakened in intensity, and a new absorption peak appears at 1650 cm^−1^, which belongs to the N-H bending vibration absorption peak in h_1_PDPA/PArg_12_, and at 1610 cm^−1^ belongs to the anti-symmetric absorption peak of C=O (COOH), which is weaker than the intensity in the HA spectrum, due to the protonation of the amine in the polymer forming an ionic bond with the carboxyl group in HA, all of which can prove the electrostatic interaction between HA and h_1_PDPA/PArg_12_.

**Figure 4:**
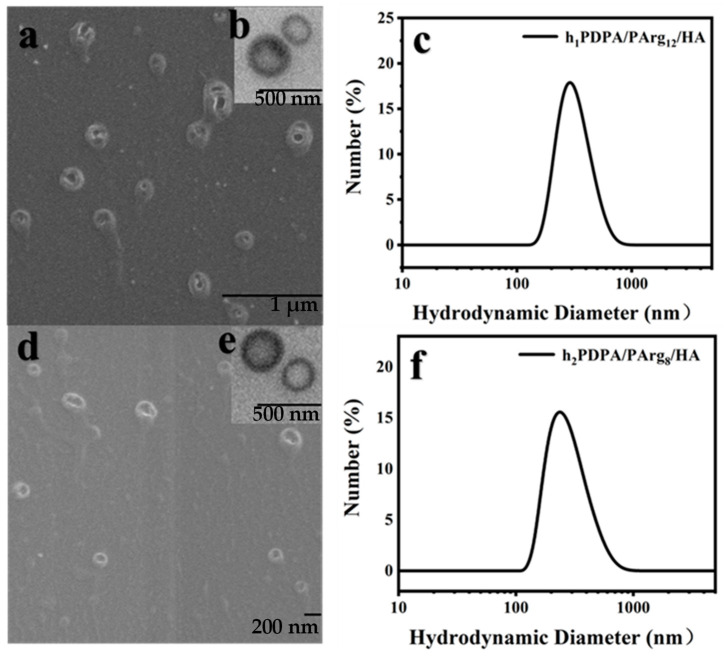
(**a**) SEM images of self-assemblies of h_1_PDPA/PArg_12_/HA at 25 °C in PB (pH = 7.4, 10 mM); (**b**) TEM images of self-assemblies of h_1_PDPA/PArg_12_/HA at 25 °C in PB; (**c**) DLS results of self-assemblies of h_1_PDPA/PArg_12_/HA at 25 °C in PB; (**d**) SEM images of self-assembly of h_2_PDPA/PArg_8_/HA at 25 °C in PB; (**e**) TEM images of self-assemblies of h_2_PDPA/PArg_8_/HA at 25 °C in PB; (**f**) DLS results of self-assemblies of h_2_PDPA/PArg_8_/HA at 25 °C in PB.

Hyperbranched polymers and their conjugates are pH sensitive due to the presence of amino groups [43]. The polymer peptide conjugates hPDPA/PArg/HA and contains both hydrophobic group DPA and hydrophilic groups PArg and HA; thus, self-assembly can be performed in PB at pH = 7.4. The self-assembly behavior of hPDPA/PArg/HA was investigated by DLS, SEM, and TEM, and the hydrodynamic dimensions of the assemblies in an aqueous solution after dialysis were measured and summarized in Table 4. The DLS results are shown in Figure 4c,f. All the two hPDPA/PArg/HA self-assemble into narrowly dispersed assemblies. Although the two hybrids are similar in composition, the hydrodynamic dimensions are slightly different. The hyperbranched polymer h_1_PDPA contains more adamantane groups, which are then recognized with more PArg, and is therefore more charged, with more HA adsorbed by electrostatic interaction and a large hydrodynamic diameter. To further investigate the morphology and structure of the assemblies, the specific morphology was first observed by SEM, which samples were prepared by dropping the assemblies’ solution on a silicon wafer and drying at 25 °C. The samples were treated with gold spray before measurement. It can be seen from the SEM images (Figure 4a,d) that both h_1_PDPA/PArg_12_/HA and h_2_PDPA/PArg_8_/HA are assembled into hollow structures with folds on the surface, indicating that they can self-assemble in an aqueous solution to form vesicles with slightly smaller sizes than those measured by DLS, suggesting that the assemblies shrink slightly during the drying process. At the same time, TEM was used to study morphology more visually. The samples were stained by OsO_4_, and it can be seen in the TEM images (Figure 4b,e) that both h_1_PDPA/PArg_12_/HA and h_2_PDPA/PArg_8_/HA self-assembled into spherical structures with low intermediate lining and high edge lining, and the sizes were in the range of 250–400 nm and 250–350 nm, respectively. The sizes were in the range of 250–400 nm and 250–350 nm, respectively, which were close to the results measured by DLS.

By using the hyperbranched polymer bioconjugates h_2_PDPA/PArg_8_/HA loaded with β-lapa. The UV absorption of a series of concentrations of β-lapa in DMSO was measured by a UV spectrophotometer, the standard curve was plotted (Figure S8), and the encapsulation rate and drug loading rate were calculated. The size and PDI of the loaded nanoparticles were measured by DLS (Figure S9). h_1_PDPA/PArg_12_/HA-β-lapa and h_2_PDPA/PArg_8_/HA-β-lapa loading data were summarized as shown in Table 5, both of which had narrower PDI, higher encapsulation rate, and lower loading due to the higher mass of adsorbed HA.

**Figure 5:**
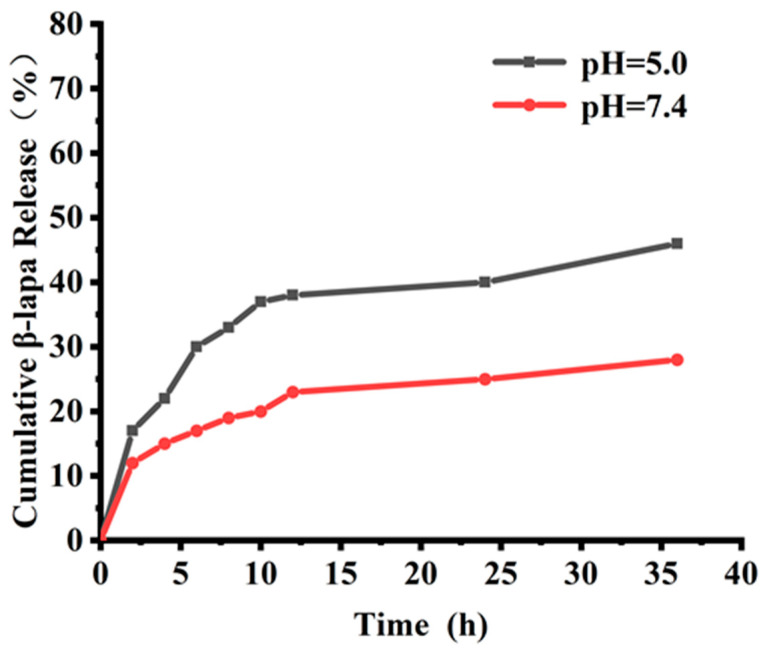
Release profile of β-lapa in h_2_PDPA/PArg_8_/HA-β-lapa.

To explore the release of β-lapa from h_2_PDPA/PArg_8_/HA-β-lapa in intracellular, two different pHs, which are 7.4 and 5.0, were selected to simulate the physiological environment of normal tissue cells and tumor cells in vitro to test the release of β-lapa under different pH conditions. As shown in Figure 5, it can be clearly seen that the release of β-lapa becomes faster and increases when the pH decreases from the physiological environment of 7.4 to the tumor microenvironment of 5.0. The cumulative release rates of the bioconjugates h_2_PDPA/PArg_8_/HA-β-lapa at pH 7.4 and 5.0 for 36 h were 28% and 48%, respectively. Its drug release rate was low in normal tissue pH conditions, while it was able to release drug molecules effectively in the low pH conditions of tumor cells.

**Figure 6:**
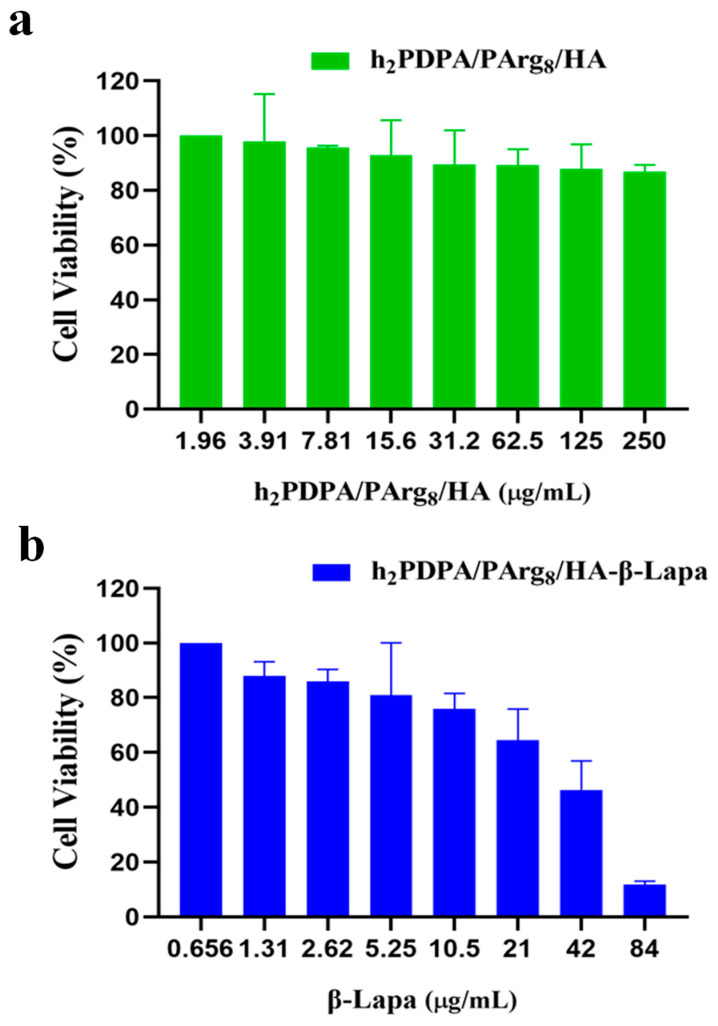
(**a**) cytotoxicity assay of h_2_PDPA/PArg_8_/HA with Hela cells after 24 h of incubation at carrier concentrations of 1.96–250 μg/mL; (**b**) cytotoxicity assay of h_2_PDPA/PArg_8_/HA-β-lapa with Hela cells after 24 h of incubation at β-lapa concentrations of 0.656–84 μg/mL.

The carriers applied for intracellular drug delivery must be non-toxic or have low toxicity, which is a necessary condition for the biological application of the assemblies. The cytotoxicity of the carrier h_2_PDPA/PArg_8_/HA on Hela cells was evaluated by using the CCK-8 method in the concentration range of 1.96–250 μg/mL and the same concentration of the carrier containing β-lapa at 0.656–84 μg/mL. It is clear from Figure 6 that the cell survival rate was above 90% at carrier concentrations up to 250 μg/mL, demonstrating that the carrier h_2_PDPA/PArg_8_/HA has low toxicity to Hela cells. The drug β-lapa was released from h_2_PDPA/PArg_8_/HA-β-lapa when in a cancer cell environment with a pH of approximately 5.0, and when the β lapa at 84 μg/mL, the cell survival rate was only 10%, demonstrating that the oxidative stress induced by β-lapa has an inhibitory effect on cancer cells.

**Figure 7:**
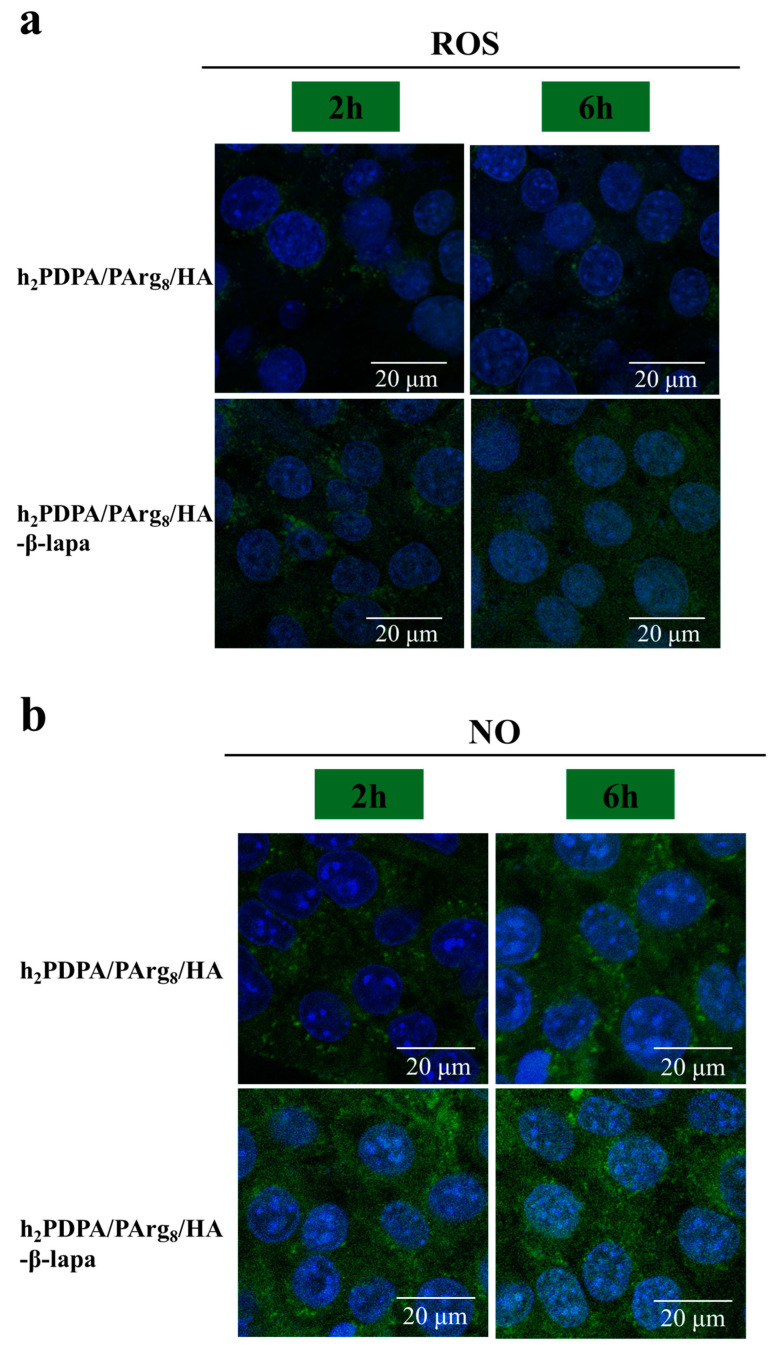

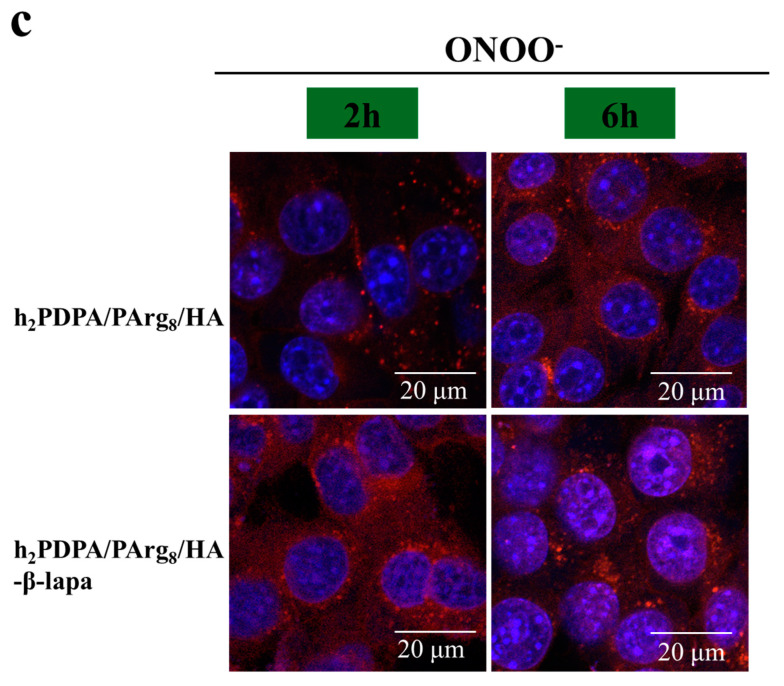
(**a**) Fluorescence superimposed images of ROS release in Hela cells treated with h_2_PDPA/PArg_8_/HA and h_2_PDPA/PArg_8_/HA-β-lapa, respectively, stained with DCFH-DA (green) for ROS and DAPI (blue) for nuclei at different time points (2 h, 6 h); (**b**) Fluorescence superimposed images of NO release in Hela cells treated with h_2_PDPA/PArg_8_/HA and h_2_PDPA/PArg_8_/HA-β-lapa, respectively, stained with DAF-FM DA (green) for NO and DAPI (blue) for nuclei at different time points (2 h, 6 h); (**c**) Fluorescence superimposed images of ONOO^−^ release in Hela cells treated with h_2_PDPA/PArg_8_/HA and h_2_PDPA/PArg_8_/HA-β-lapa, respectively, stained with O72 (red) for ONOO^−^ and DAPI (blue) for nuclei at different time points (2 h, 6 h).

The h_2_PDPA/PArg_8_/HA and h_2_PDPA/PArg_8_/HA-β-lapa were co-cultured with Hela cells, respectively, and the release of ROS was observed by laser confocal microscopy (CLSM) at different time points. The nuclei were stained blue with DAPI, and ROS production in Hela cells was detected by ROS probe (DCFH-DA), as shown in Figure 7a. The green fluorescence in h_2_PDPA/PArg_8_/HA and h_2_PDPA/PArg_8_/HA-β-lapa was significantly enhanced with increasing time, demonstrating the production of ROS due to the shell of the polymer hybrid material is biocompatible and targeted HA, making the nanoparticles easily internalized by the cells. Since the hyperbranched polymer is pH-responsive, the green fluorescence of h_2_PDPA/PArg_8_/HA-β-lapa is stronger than that of unloaded h_2_PDPA/PArg_8_/HA due to the release of β-lapa in the tumor environment (acidic), which can generate large amounts of H_2_O_2_. PArg can be used as a NO donor with good biocompatibility, and in the presence of large amounts of ROS conditions, NO was released in a controlled manner. As shown in Figure 7b, the green fluorescence in h_2_PDPA/PArg_8_/HA and h_2_PDPA/PArg_8_/HA-β-lapa was significantly enhanced with increasing time, demonstrating the production of NO. The green fluorescence in h_2_PDPA/PArg_8_/HA-β-lapa was stronger than that in h_2_PDPA/PArg_8_/HA due to the large amount of ROS that can oxidize PArg to produce NO. Because H_2_O_2_ can penetrate most cell membranes and generate hydroxyl radical ·OH with intracellular Fe, ·OH and NO can generate peroxynitrite anion (ONOO^−^), and the generation of ONOO^−^ in cells was detected by the O72 probe. The red fluorescence in h_2_PDPA/PArg_8_/HA and h_2_PDPA/PArg_8_/HA-β-lapa was significantly enhanced with increasing time, demonstrating the production of ONOO^−^ (Figure 7c). The red fluorescence is stronger in h_2_PDPA/PArg_8_/HA-β-lapa than in h_2_PDPA/PArg_8_/HA because of the high production of ROS and thus more ONOO^−^.

## 4. Conclusions

First, a monomer initiator ABMA with double bonds, bromine groups, and adamantane groups was obtained by a Passerini three-component reaction using CHO-Br, Ad-NC, and methacrylic acid. The inimer was copolymerized with the monomer DPA, and pH-responsive hyperbranched polymer hPDPA with different DB were prepared by the ATRP-SCVP method. The pH-responsive hyperbranched polymer peptide conjugates hPDPA/PArg were obtained by the molecular recognition of adamantyl groups on hyperbranched polymers with β-CD-PArg, and then the pH-responsive polymer peptide hybrid material hPDPA/PArg/HA was obtained by the electrostatic interaction between hPDPA/PArg and HA. The self-assembly behavior of hPDPA/PArg/HA with different DB and the application of the assemblies as drug carriers for β-lapa were investigated. The two hybrid materials h_1_PDPA/PArg_12_/HA and h_2_PDPA/PArg_8_/HA were found to self-assemble to form vesicles in PB at pH = 7.4, and β-lapa can be effectively loaded by vesicles and released under acidic conditions (pH = 5.0). The carriers h_2_PDPA /PArg_8_/HA showed low cytotoxicity towards Hela cells. The release of β-lapa leads to an increase in reactive oxygen species, which can react with PArg to generate NO. The reaction between reactive oxygen species and NO leads to the production of ONOO^−^, and this synergistic therapy based on ROS and NO has a significant inhibitory effect on cancer cells.

## Figures and Tables

**Table 1:** Polymerization formulations of hyperbranched polymers hPDPA.

	DPA/mg	ABMA/mg	CuBr/mg	bpy/mg	DMF/mL	Time/h
h_1_PDPA	300	123	17.1	55.9	0.4	5
h_2_PDPA	300	72.8	10.1	33.0	0.4	5

**Table 2:** Summary of assembly data of two polymer hybrid materials.

	hPDPA/mg	β-CD-PArg/mg	HA/mg
h_1_PDPA	0.250	0.307	16.0
h_2_PDPA	0.250	0.218	16.0

**Table 3:** Structure data of the hyperbranched polymers.

	h_1_PDPA_12_	h_2_PDPA_8_
Feed ratio of DPAMA to ABMA	6	10
DP_n_ of ABMA	12	8
DP_n_ of DPAMA	70	90
*M*_n_ (kDa) ^a^	21.1	23.3
PDI	1.57	1.56
DB ^b^	0.225	0.156

^a^: The *M*_n_ was determined according to the ^1^H NMR result. ^b^: DB = 2D/(2D + L) D: integral area of the branching units L: integral area of the linear units.

**Table 4:** Structure data of the two bioconjugates and DLS results of their self-assemblies.

	D_h_ (nm)	PDI
h_1_PDPA_12_/PArg/HA	330	0.107
h_2_PDPA_8_/PArg/HA	280	0.145

**Table 5:** The drug loading data of the two polymer hybrid materials were summarized at 25 °C.

	D_h_ (nm)	PDI	EE/%	LC/%
h_1_PDPA	395	0.126	67.7	1.05
h_2_PDPA	330	0.143	60.8	1.54

## Data Availability

The data presented in this study are available on request from the corresponding author.

## Supplementary Materials

The following supporting information can be downloaded at: https://www.mdpi.com/article/10.3390/nano13111725/s1. Figure S1: ^1^H NMR and ^13^C NMR spectra of 1-adamantyl isocyanide (Ad-NC) in CDCl_3_; Figure S2: ^1^H NMR spectra of 4-formylphenyl 2-bromo-2-methylpropanoate (CHO-Br) in CDCl_3_; Figure S3: ^1^H NMR and ^13^C NMR spectra of 2-((adamantan-1-yl)amino)-1-(4-((2-bromo-2-methylpropanoyl)oxy)phenyl)-2-oxoeth yl methacrylate (ABMA) in CDCl_3_; Figure S4: ^1^H NMR spectrum of h_2_PDPA in CDCl_3_; Figure S5: GPC curves of h_2_PDPA; Figure S6: ^1^H NMR spectrum of β-CD-dithiopyridine in DMSO; Figure S7: ^1^H NMR spectrum of (a) PArg and (b) β-CD-PArg in DMSO-d_6_; Figure S8: (a) UV absorption of different concentrations of β-lapa in DMSO; (b) the standard curve of β-lapa; (c) UV absorption of the assembly in DMSO solution; Figure S9: Hydrodynamic dimensional drawing of h_1_PDPA/PArg_12_/HA-β-lapa (black line) and h_2_PDPA/PArg_8_/HA-β-lapa (red line) assemblies at 25 °C.
