# Supplementary material for: Preparation and Self-Assembly of pH-Responsive Hyperbranched Polymer Peptide Hybrid Materials
Source: Nanomaterials (Basel). 2023 May 25;13(11):1725. doi: 10.3390/nano13111725 (PMC10254404; doi:10.3390/nano13111725)
Supplement: Supplementary file 1 [file nanomaterials-13-01725-s001.zip › nanomaterials-2405073-supplementary.pdf]

Supporting Information

# Preparation and self-assembly of pH-responsive hyperbranched polymeric peptide hybrid materials

Yue Zhang*, Yan Qin, Jianguo Yi

**Characterization**: $^1$H NMR measurements were carried out on a 400 MHz Varian UNITY-plus NMR spectrometer. The $M_n$ and the dispersity were measured by a Hitachi gel permeation chromatography (GPC) with THF as the mobile phase and PMMA as the standards. The GPC was equipped with a Waters 2487 dual wavelength UV detector and a Waters 2414 differential refractive index detector. Scanning electron microscopy (SEM) was measured with an FEI Apreo S LoVac electron microscope. The hydrodynamic diameters ($D_h$), the polydispersities (PDI) of the assemblies and pH responsiveness of hyperbranched polymers and biocouples were measured by a Malvern Zetasizer Nano-ZS. The drug encapsulation rate and drug loading capacity were determined on a Shimadzu UV-2450 spectrophotometer. Electrostatic interactions between biocouplers were determined on a Bruker TENSOR II Fourier transform infrared spectrometer. Cellular ROS and NO release images were observed on an OLYMPUS FV 1200 laser confocal microscope.

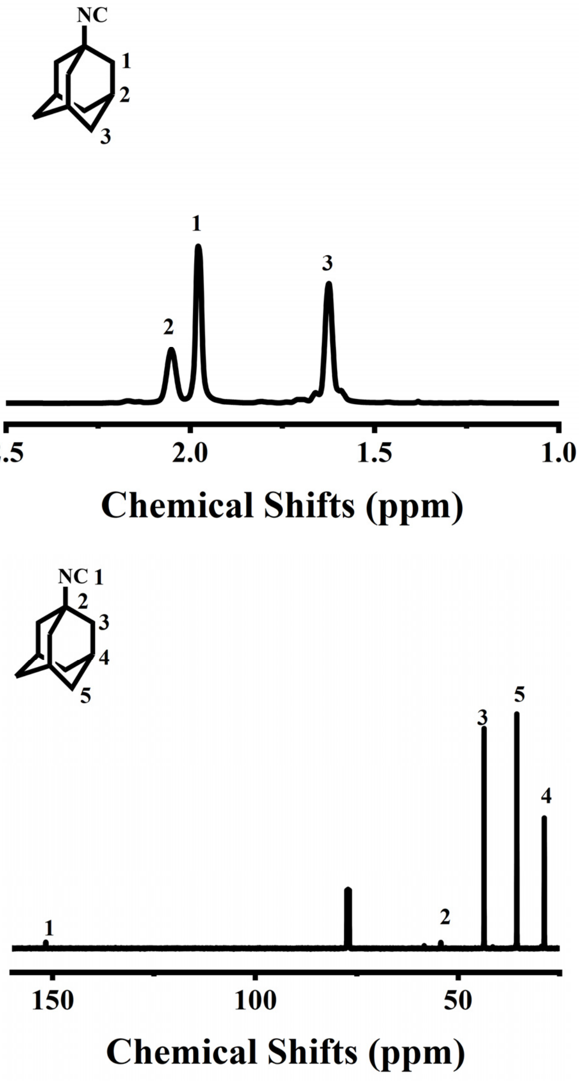


**Figure S1.** $^1$H NMR and $^{13}$C NMR spectra of 1-adamantyl isocyanide (Ad-NC) in $CDCl_3$.

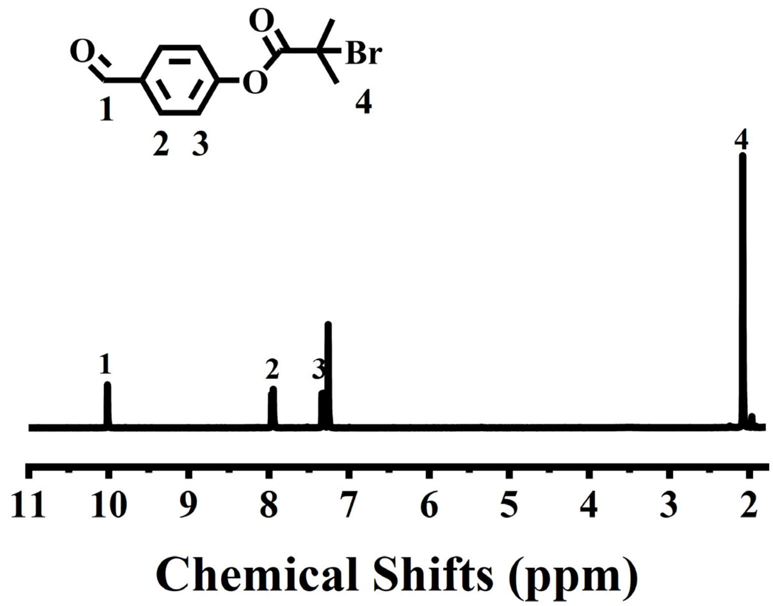


**Figure S2.** $^{1}$H NMR spectra of 4-formylphenyl 2-bromo-2-methylpropanoate (CHO-Br) in $CDCl_3$.

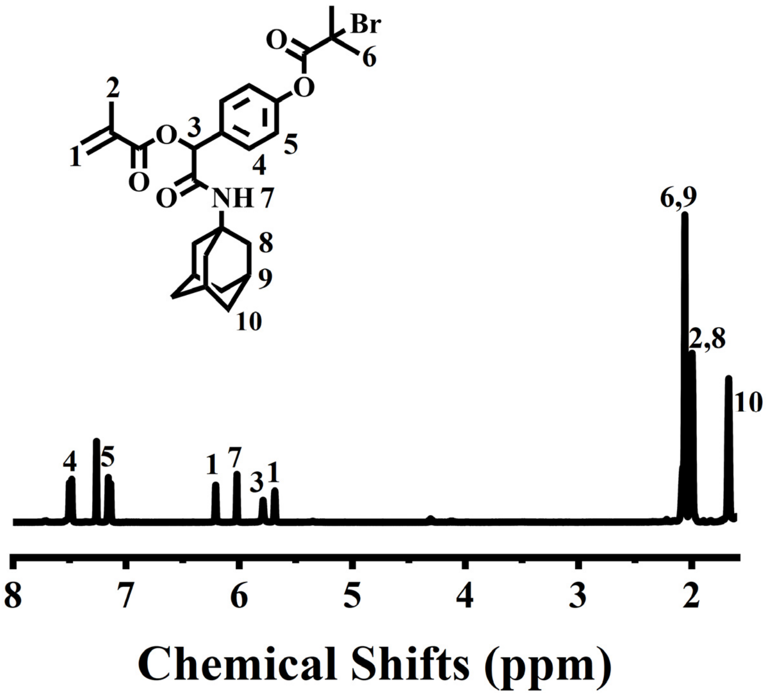

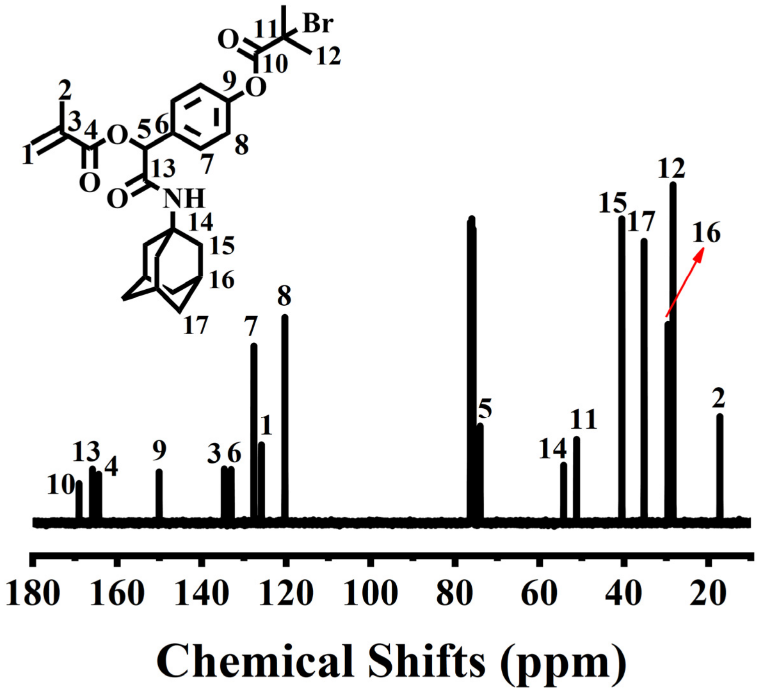


**Figure S3.** $^1$H NMR and $^{13}$C NMR spectra of 2-((adamantan-1-yl)amino)-1-(4-((2-bromo-2-methylpropanoyl)oxy)phenyl)-2-oxoeth yl methacrylate (ABMA) in $CDCl_3$.

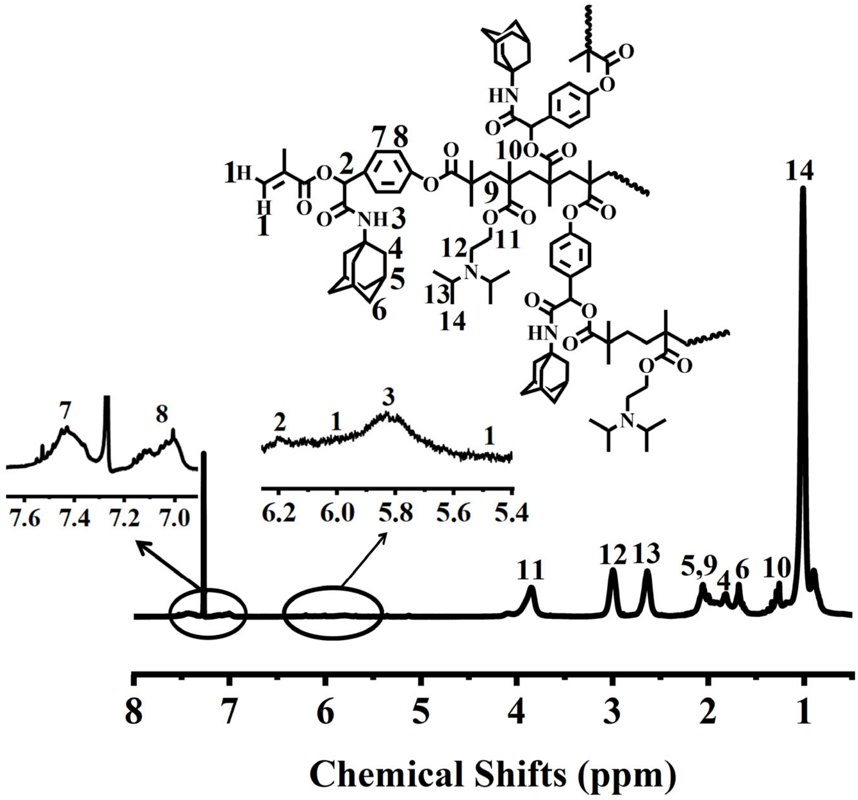


**Figure S4.** $^1$H NMR spectrum of $h_2$PDPA in $CDCl_3$.

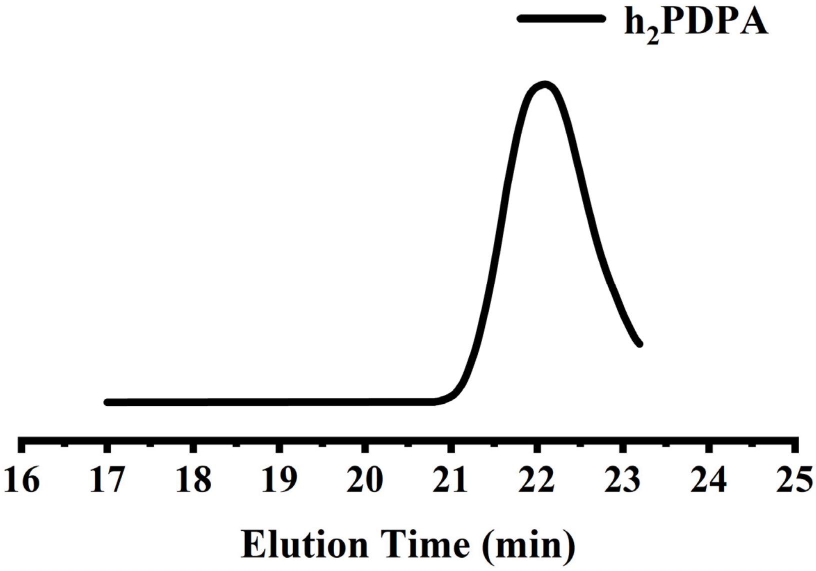


**Figure S5.** GPC curves of $h_2$PDPA.

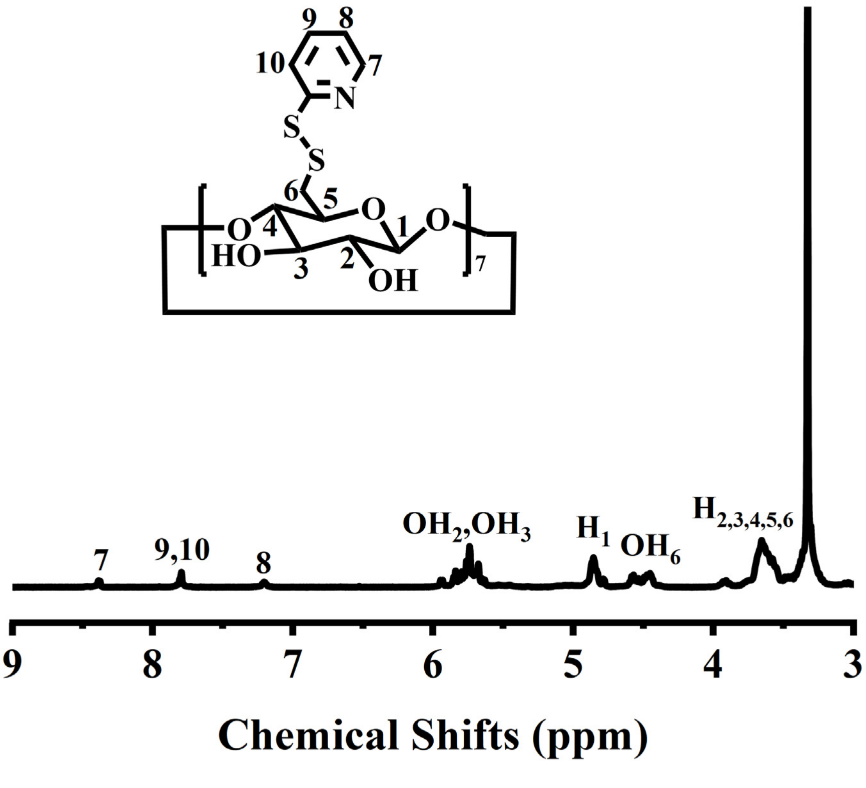


**Figure S6.** $^1$H NMR spectrum of β-CD-dithiopyridine in DMSO.

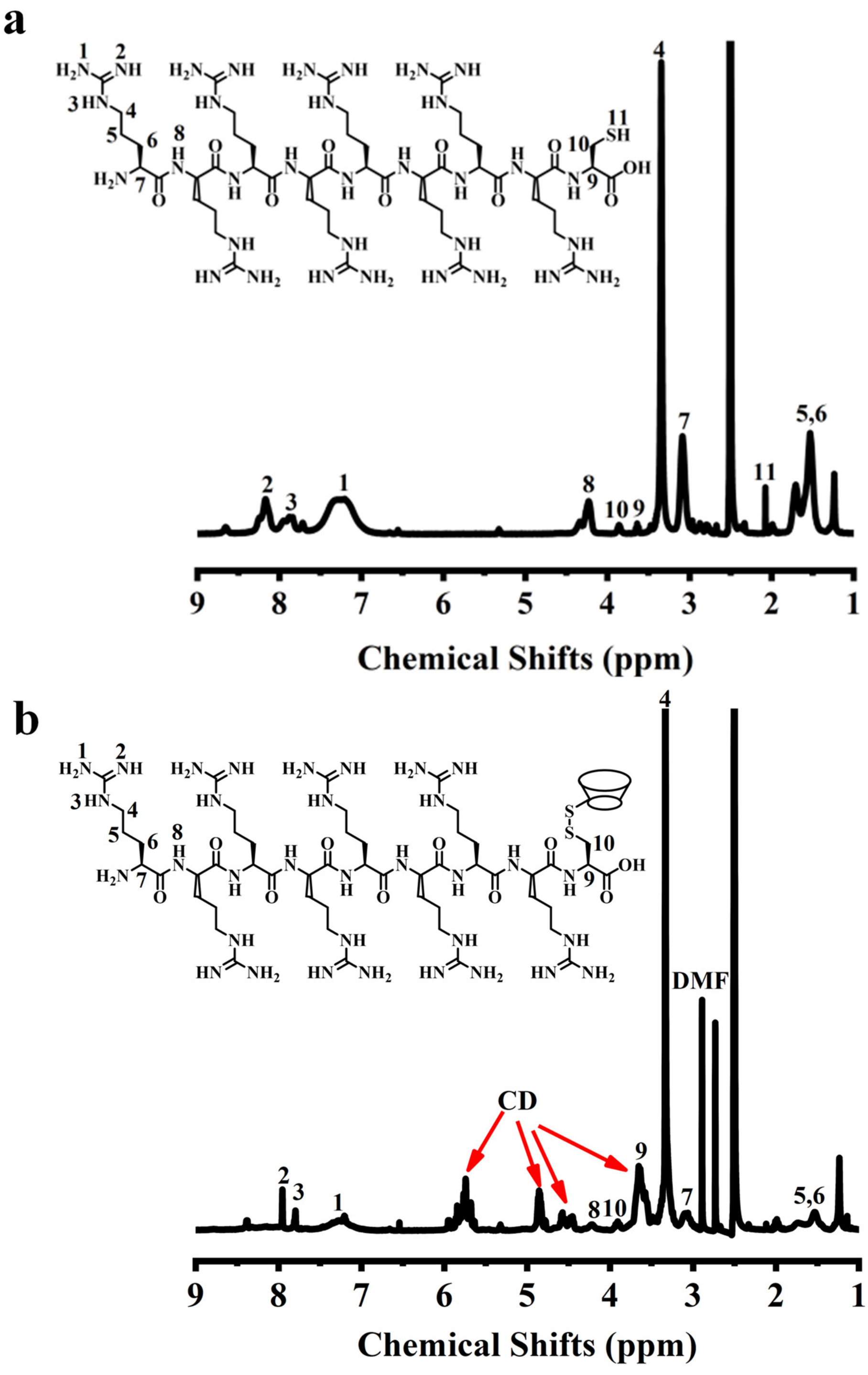


**Figure S7.** $^{1}$H NMR spectrum of (a) PArg and (b) β-CD-PArg in DMSO-$d_6$.

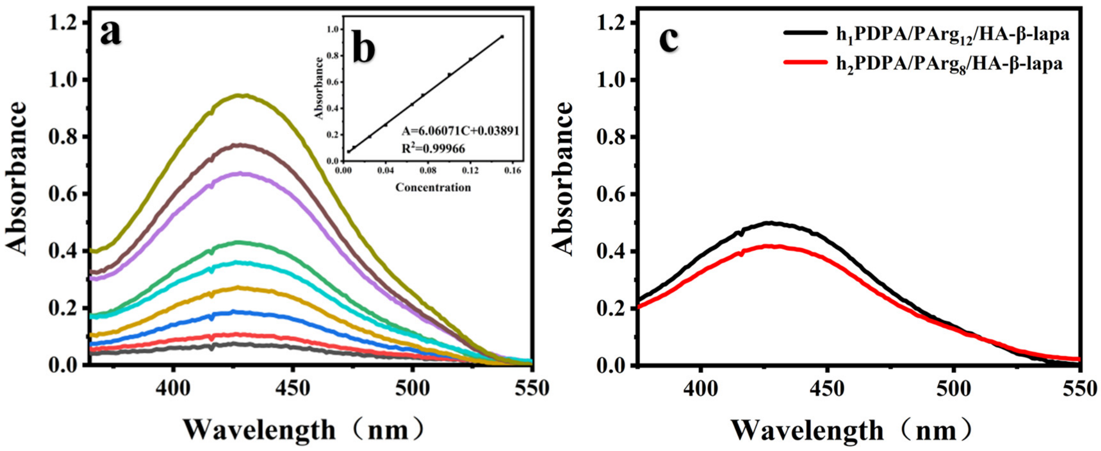


**Figure S8.** (a) UV absorption of different concentrations of β-lapa in DMSO; (b) the standard curve of β-lapa; (c) UV absorption of the assembly in DMSO solution.

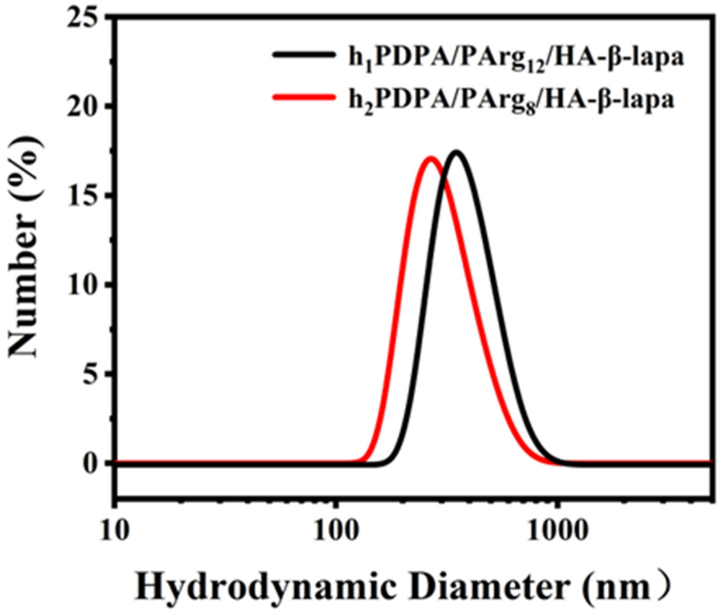


**Figure S9.** Hydrodynamic dimensional drawing of $h_1$PDPA/PArg$_{12}$/HA-β-lapa (black line) and $h_2$PDPA/PArg$_8$/HA-β-lapa (red line) assemblies at 25°C.
